# Vestibular Migraine in Children and Adolescents: Clinical Findings and Laboratory Tests

**DOI:** 10.3389/fneur.2014.00292

**Published:** 2015-01-26

**Authors:** Thyra Langhagen, Nicole Lehrer, Ingo Borggraefe, Florian Heinen, Klaus Jahn

**Affiliations:** ^1^German Center for Vertigo and Balance Disorders, Ludwig-Maximilians-University of Munich, Munich, Germany; ^2^Department of Pediatric Neurology and Developmental Medicine, Ludwig-Maximilians-University of Munich, Munich, Germany; ^3^Schön Klinik Bad Aibling, Bad Aibling, Germany

**Keywords:** migraine-related vertigo, vestibular migraine, somatoform vertigo, ocular motor signs

## Abstract

**Introduction:** Vestibular migraine (VM) is the most common cause of episodic vertigo in children. We summarize the clinical findings and laboratory test results in a cohort of children and adolescents with VM. We discuss the limitations of current classification criteria for dizzy children.

**Methods:** A retrospective chart analysis was performed on 118 children with migraine related vertigo at a tertiary care center. Patients were grouped in the following categories: (1) definite vestibular migraine (dVM); (2) probable vestibular migraine (pVM); (3) suspected vestibular migraine (sVM); (4) benign paroxysmal vertigo (BPV); and (5) migraine with/without aura (oM) plus vertigo/dizziness according to the International Classification of Headache Disorders, 3rd edition (beta version).

**Results:** The mean age of all patients was 12 ± 3 years (range 3–18 years, 70 females). 36 patients (30%) fulfilled criteria for dVM, 33 (28%) for pVM, 34 (29%) for sVM, 7 (6%) for BPV, and 8 (7%) for oM. Somatoform vertigo (SV) co-occurred in 27% of patients. Episodic syndromes were reported in 8%; the family history of migraine was positive in 65%. Mild central ocular motor signs were found in 24% (most frequently horizontal saccadic pursuit). Laboratory tests showed that about 20% had pathological function of the horizontal vestibulo-ocular reflex, and almost 50% had abnormal postural sway patterns.

**Conclusion:** Patients with definite, probable, and suspected VM do not differ in the frequency of ocular motor, vestibular, or postural abnormalities. VM is the best explanation for their symptoms. It is essential to establish diagnostic criteria in clinical studies. In clinical practice, however, the most reasonable diagnosis should be made in order to begin treatment. Such a procedure also minimizes the fear of the parents and children, reduces the need to interrupt leisure time and school activities, and prevents the development of SV.

## Introduction

Migraine-related syndromes [such as benign paroxysmal vertigo (BPV) and vestibular migraine (VM)] are the most common cause of episodic vertigo in children (1–6). Of the children and adolescents who present with dizziness, 35–60% also have headache, which can precede, follow, or occur simultaneously with vestibular symptoms (7, 8). The term VM was first established on the basis of clinical observation and epidemiological data of adults (9, 10). The diagnostic criteria for VM proposed by Neuhauser et al. (11) were recently validated (10, 12) and published in a consensus document of the Bárány Society and the International Headache Society (13). Our study posed the following questions: (a) What percentage of all patients who clinically have migraine-related vertigo can be classified as having VM? (b) Do patients who fulfill the criteria for VM differ from patients who do not in terms of clinical characterization or results of laboratory tests?

## Materials and Methods

A retrospective chart analysis was performed on data of children and adolescents who were referred to the German Center for Vertigo and Balance Disorders between 11/2009 and 05/2014 and been suspected to have VM at the first visit. The database included patient demographics, diagnosis, neuro-ophthalmological, and neurological status, as well as laboratory test results (ocular motor testing, video head impulse test, caloric irrigation, subjective visual vertical (SVV), static posturography) in the symptom-free interval. For classification, we used the recently defined criteria of the International Classification of Headache Disorders, 3rd edition (beta version) (ICHD-3 beta) and the Bárány Society adapted for children (14).

The following diagnostic groups were established: (a) (definite) vestibular migraine (dVM) according to defined criteria (13), (b) probable vestibular migraine (pVM) according to defined criteria, (c) suspected vestibular migraine (sVM) but not fulfilling defined criteria, (d) BPV according to ICHD-3 beta, and (e) other migraine syndromes (oM) with or without aura according to ICHD-3 beta, including brainstem aura who presented with dizziness or vertigo (Table 1).

**Table 1:** **Diagnostic groups in migraine-related vertigo**.

Definite vestibular migraine (dVM)	Probable vestibular	Suspected vestibular	Benign paroxysmal	Other migraines with/without
	migraine (pVM)	migraine (sVM)	vertigo (BPV)	aura plus vertigo/dizziness (oM)
**A**. At least 5 episodes with vestibular symptoms of moderate or severe intensity, lasting 5 min to 72 h	**A**. At least 5 episodes with vestibular symptoms of moderate or severe intensity, lasting 5 min to 72 h	**A**. At least 2 episodes with vestibular symptoms of moderate or severe intensity lasting seconds to 72 h	**A**. At least 5 attacks fulfilling criteria B and C	**A**. To fulfill the diagnostic criteria for migraine with aura, migraine without aura or migraine with brainstem aura
**B**. Current or previous history of migraine with or without aura according to ICHD-3 beta	**B**. Only one of the criteria B and C for dVM is fulfilled (migraine history *or* migraine features during the episode)	**B**. One or more migraine features with at least 50% of the vestibular episodes:	**B**. Vertigo occurring without warning, maximal at onset and resolving spontaneously after minutes to hours without loss of consciousness	**B**. Vestibular symptoms that do not fulfill the criteria for VM (A.) or which are caused by an associated vestibular disorder
		• headache with at least two of the following characteristics: one sided location, pulsating quality, moderate or severe pain intensity, aggravation by routine physical activity
		• photophobia or phonophobia
		• visual or other aura
**C**. One or more migraine features with at least 50% of the vestibular episodes:	**C**. Not better accounted for by another vestibular or ICHD-3 beta diagnosis	**C**. Not better accounted for by another vestibular or ICHD-3 beta diagnosis	**C**. At least one of the following associated symptoms or signs:	
• headache with at least two of the following characteristics: one sided location, pulsating quality, moderate or severe pain intensity, aggravation by routine physical activity			
• photophobia and phonophobia
• visual aura
			• nystagmus
			• ataxia
			• vomiting
			• pallor
			• fearfulness
**D**. Not better accounted for by another vestibular or ICHD-3 beta diagnosis			**D**. Normal neurological examination and audiometric and vestibular functions between attacks	
			**E**. Not attributed to another disorder.	

*Criteria are based on the International Classification of Headache Disorders, 3rd edition (beta version)*.

*ICHD-3 beta, International Classification of Headache Disorders, 3rd edition (beta version); dPV, definite vestibular migraine; pVM, probable vestibular migraine; sVM, suspected vestibular migraine; BPV, benign paroxysmal vertigo; oM, other migrain syndromes*.

### Associated somatoform vertigo

Associated somatoform vertigo (SV) was suspected on the basis of criteria suggested by Eckhardt-Henn and Dieterich (15, 16). SV is defined to be a psychosomatic disorder associated with different psychiatric comorbidities (anxiety disorders, depressive, dissociative, and somatoform disorders) (16). An additional psychological interview was not routinely performed.

### Orthostatic dizziness

Orthostatic dizziness was diagnosed clinically if a short (seconds to 5 min) non-rotational dizziness, light-headedness, feeling of impending blackout, or faint was provoked when arising from a supine or sitting position (17, 18).

### Ocular motor findings

Vertical saccadic pursuit was always considered as normal, and horizontal saccadic pursuit was considered as normal until the age of 7 years (19, 20).

### Laboratory testing

Bithermal caloric testing was considered pathological if the side-to-side difference was greater than 25% (Jongkees’s formula), and/or peak slow phase velocity was <5°/s, or if there was a directional preponderance greater than 30%. The SVV was regarded as abnormal if there was a deviation greater than 2.5° in the static measurement (21). The video head-impulse test (vHIT) was pathological if there were open or covered saccades, or a reduced gain (<0.7) (22, 23). Static posturography was measured with the Kistler platform and considered pathological if postural sway was increased or showed pathological patterns in the neural net (own normative data) (24). Cervical vestibular evoked myogenic potentials (cVEMP) were recorded on the sternocleidomastoid muscles and were pathological with reduced amplitude or if absent.

As this was a descriptive retrospective study and the patients were not comparably distributed, we present only percentages and did not apply statistical analysis.

## Results

### Patients

The mean age of the patients was 12 ± 3 years (range 3–18 years, 70 females). The girls’ age ranged from 3 to 17 years with a mean age of 12.4 ± 3 years, and the boys’ age ranged from 3 to 18 years with a mean age of 12.9 ± 3 years. Data on the ages and gender in the different diagnostic groups are given in Table 2.

**Table 2:** **Patient’s distributions among the diagnostic groups**.

	dVM	pVM	sVM	BPV	oM	All patients
Number of patients	36	33	34	7	8	118
% (*n* = 118)	30%	28%	29%	6%	7%	100%
Age (years)	13.3 ± 2	12.7 ± 3	13.3 ± 2	4.2 ± 1	13.4 ± 2	12 ± 3
Range (years)	7–17	6–17	8–18	3–6	9–16	3–18
Girls (*n* = 70)	31%	27%	27%	9%	6%	100%
Boys (*n* = 48)	29%	29%	31%	2%	9%	100%

*dPV, definite vestibular migraine; pVM, probable vestibular migraine; sVM, suspected vestibular migraine; BPV, benign paroxysmal vertigo; oM, other migraine syndromes*.

### Diagnosis

Thirty percent of 118 patients fulfilled the diagnostic criteria for dVM, 28% for pVM, 29% for sVM, 6% were diagnosed to have BPV, and 7% oM (without aura *n* = 4, with aura *n* = 3, brainstem aura *n* = 1).

### Clinical characterization

Eighty-six patients (73%) reported associated headache in all or some of their vertigo attacks. Twenty-two of these patients also complained of headache attacks without vertigo, 17 (14%) reported only headache independently of vertigo attacks, and 15 (13%) reported no headache at all. All patients with dVM had migraine headache as defined in the diagnostic criteria. In the group of patients with pVM, 18% never mentioned having a headache, but presented with the other migraine features in at least half of their vertigo attacks (phono- and photophobia or visual aura). Most of the patients (82%) with pVM reported headaches, but did not fulfill the diagnostic criteria for migraine. In the group of sVM, 3 patients could not be classified as pVM because they reported less than 5 episodes; 3 patients did not have attacks intense enough to interfere with activity; and 5 children had episodes that were too short (lasting only seconds). The most common reason for not fulfilling the criteria for pVM was that migraine features were not complete (20 patients, for example, had photo- but no phonophobia or *vice versa*). The family history of migraine was positive in 65% of all patients.

Recurrent abdominal pain not related to the vertigo spells was reported by 2 patients with sVM; abdominal pain presenting together with vertigo was mentioned by 7 patients (2 with dVM, 5 with pVM). Three patients had previously abdominal migraine (2 with dVM, 1 with pVM).

Eight patients also suffered from orthostatic dizziness (7%, 4 females, 4 with dVM, 3 with pVM, 1 with oM) and eight patients reported having a syncope (7%, 5 females, 4 with dVM, 2 with pM, 1 with sVM, 1 with oM).

Two patients (1 dVM, 1 pVM) complained of unilateral tinnitus during some of their vertigo attacks, but without hearing loss or other auditory symptoms; their audiometric testing was unremarkable. One child in the sVM group had previously been diagnosed to have mild sensorial hearing loss, but did not mention tinnitus, hearing loss, or pressure in the ears during the attacks.

Associated SV was suspected in 27% of the children (26% of the girls, 29% of the boys). The mean age of children with associated SV was 13.8 ± 2 years (range 8–17 years), the mean age of children without SV was 12.2 ± 3 years (range 3–18 years). The co-occurrence of SV in the different diagnostic groups is shown in Table 3.

**Table 3:** **Clinical features of the diagnostic groups**.

	dVM	pVM	sVM	BPV	oM	All patients
Headache (%)	100	82	85	37	100	73
Associated SV (%)	17	42	35	0	0	27
Abdominal pain (%)	6	15	6	0	0	8
Orthostatic dizziness (%)	11	9	0	0	12	7
Syncope (%)	11	6	3	0	12	7
Tinnitus (%)	3	3	0	0	0	2

*dPV, definite vestibular migraine; pVM, probable vestibular migraine; sVM, suspected vestibular migraine; BPV, benign paroxysmal vertigo; oM, other migraine syndromes; SV, somatoform vertigo*.

Nine patients [8% if children with BPV were excluded (*n* = 111)] had previously had episodic syndromes that may have been associated with migraine (3 abdominal migraine, 3 BPV, 2 cyclic vomiting, 1 torticollis).

Data on motion sickness were available for 53 patients; 27 patients (51%) reported having had car sickness.

### Ocular motor signs and laboratory testing

At least mild central ocular motor signs (COMS) were observed in 24% of all patients (25% with dVM, 21% with pVM, 29% with sVM, 12% with oM (Table 4).

**Table 4:** **Central ocular motor signs in migraine-related vertigo**.

% (*n*)	dVM	pVM	sVM	BPV	oM	All patients
Presence of central ocular motor signs	25% (10)	21% (7)	29% (10)	0% (1)	12% (1)	27% (28)
Saccadic pursuit	24% (8)	18% (6)	21% (7)	0% (1)	0% (1)	18% (21)
Gaze evoked nystagmus	6% (3)	6% (2)	6% (2)	0% (1)	12% (1)	6% (7)
Upbeat nystagmus (funduscopy)	6% (2)	0% (1)	3% (1)	0% (1)	0% (1)	2% (3)
Ocular flutter	3% (1)	0% (1)	0% (1)	0% (1)	0% (1)	1% (1)
Impaired suppression of VOR	0% (1)	3% (1)	6% (2)	0% (1)	0% (1)	2% (3)
Downbeat nystagmus provoked by headshaking	0% (1)	0% (1)	3% (1)	0% (1)	0% (1)	1% (1)

*dPV, definite vestibular migraine; pVM, probable vestibular migraine; sVM, suspected vestibular migraine; BPV, benign paroxysmal vertigo; oM, other migrain syndromes; VOR, vestibular ocular reflex*.

Bithermal caloric testing was pathological in 21% of all examined patients. The SVV was abnormal (tilted > 2.5°) in 11%. The video head-impulse test was abnormal in 19%; cervical vestibular evoked myogenic potentials (cVEMP) were remarkable in 38%. Posturography was abnormal in 48% of patients (including paradoxical improvement with more difficult tasks in SV). The results of laboratory tests are summarized in Table 5. An MRI was performed in 52% of the patients; all findings were not pathological.

**Table 5:** **Results of the laboratory tests**.

% *(n*)	dVM	pVM	sVM	BPV	oM	All patients
Pathologic caloric testing	21%	16%	29%	25%	0%	21% (*n* = 100)
SVV deviation	16%	3%	12%	20%	0%	11% (*n* = 113)
Pathologic vHIT	8%	20%	29%	50%	0%	19% (*n* = 70)
Pathologic cVEMP	33%	50%	50%	67%	0%	38% (*n* = 47)
Abnormal posturography	50%	4%	62%	0%	75%	48% (*n* = 98)

*dPV, definite vestibular migraine; pVM, probable vestibular migraine; sVM, suspected vestibular migraine; BPV, benign paroxysmal vertigo; oM, other migraine syndromes; SVV, subjective visual vertical; vHIT, video head-impulse test; cVEMP, cervical vestibular evoked myogenic potentials*.

## Discussion

The diagnostic criteria for VM were defined in adults and are based on the patient clinical history. Compared to adults, children and adolescents have a shorter medical history to establish a clinical diagnosis, and depending on their age, might have difficulties exactly describing their symptoms. A group of patients (sVM, 34 patients, 29%) could not be classified as pVM, although migraine was the most reasonable explanation for their vertigo and/or dizziness. Particularly, the migraine features that should present together with the vertigo were often not fulfilled, the duration of the attacks was too short (not reaching minutes), or the number of attacks was too low. This group would have fit in the now obsolete diagnostic category of possible vestibular migraine.

### Co-occurrence of headache in vestibular disorders

Headache was reported by 73% of our patients, mostly occurring simultaneously with the vertiginous symptoms (54%). In a large retrospective study (*n* = 145) on vestibular disorders in childhood and adolescence, headache with vertigo occurred in 32% of younger patients and in 39% of adolescents (2). In a smaller retrospective study (*n* = 37), 51% of the patients younger than 18 years presented with associated headache (1). In a large epidemiological study of dizziness in 10-year-old children, a total of 60% of the children (*n* = 400) reported headaches with their dizziness (8). In a population-based study about paroxysmal vertigo in children, vertigo was reported during headache attacks by 69% of patients with migraine (*n* = 159) (25). As we focused on children with VM, it is comprehensible that our percentages of headache are higher.

### Co-occurrence of somatoform vertigo in vestibular migraine

We observed a co-occurrence with SV in 27% of the children. The co-occurrence of SV and VM was shown in a previous study (26). In that study, SV was overall more common in pubescent girls older than 12 years of age (46%) (26). Several studies in adults have reported the comorbidity of balance disorders, anxiety disorders, and migraine (27–32). Functional alterations in the brain of migraineurs as well as interoceptive and cognitive adaptations might cause this association. The pathways participating in the generation, perception, and regulation of emotions and affective states share the network of afferent loops in the brainstem and cortex for sensorimotor integration (26, 29). Eckhardt-Henn et al. reported manifest comorbid psychological disorders (anxiety, phobic disorders, and depression) in 65% of adult patients with VM (30). Another organic vestibular disorder in adults, which often presents with SV is Menière’s disease (16, 27, 30). High values for anxiety and depression were found with psychometric testing performed in patients with VM or Menière’s disease (27).

A recent study in children with episodic vertigo reported significantly higher mean scores in almost all scales of the following psychological assessments: Strength and Difficulties Questionnaire (SDQ), Depression Inventory (CDI), and Screen for Child anxiety-related Emotional disorders (SCARED), except in prosocial behavior and separation anxiety (33). A previous study on the psychological assessment of children and adolescents with BPV, migraine, and controls using the Child Behavior Checklist (CBCL), the Children’s Depression Inventory (CDI), and the Multidimensional Anxiety Scale for Children (MASC) found that BPV and VM patients had higher CBCL scores (total, internalizing, and externalizing) as well as higher CDI and MASC scores than controls (26, 34). A recent study by Lahmann et al. (31) reported that almost half of adult patients with vertigo/dizziness had a psychiatric comorbidity and showed psychosocial impairment. The development of anticipation anxiety in adult patients with migraine appears to be facilitated by the unpredictability of the vertigo attacks. A sensation of panic-like anxiety during the attacks is perceived by adult patients (30). Children and adolescents often experience vertigo as frightening, and this anxiety might be conducive to the development of SV. An early diagnosis of the vestibular syndrome is important so as to minimize the worries of both patients and parents about the origin of the symptoms and to devise a therapeutic strategy to prevent the development of somatoform complaints. On the other hand, it is important to carefully characterize earlier attacks of children presenting with SV in order to avoid overlooking a VM or BPV (26).

### Co-occurrence of orthostatic dizziness and/or syncope

Symptoms and signs of orthostatic dizziness were also reported by 7% of the children and at least one syncope by 7%. Orthostatic intolerance and syncope have been reported to be more frequent in adult patients with migraine (35), particularly in women (35, 36). The underlying physiopathology is unclear, as no gross abnormality of the autonomic nervous system could be demonstrated (35). There could be a slight sympathetic dysfunction, such as reduced plasma noradrenaline levels and alfa-adrenergic hypersensitivity, which have been reported among migraineurs (35, 37).

### Episodic syndromes that may be associated with migraine

Eight percent (9 children) of our patients with migraine (dVM, pVM, sVM, oM) had previously had an episodic syndrome that might be associated with migraine according to the ICHD-3 beta (38). One of our patients with BPV of childhood had benign paroxysmal torticollis of infancy (BPTI). BPTI has been reported to be followed by BPV of childhood (39, 40) and migraine (40, 41). Several studies reported the later development of migraine headaches in children with periodic syndromes in infancy and childhood (40, 42–44) and a genetic linkage to CACNA1A (40) and to PRRT2 (45) has been published in case series. In our experience, previous episodic syndromes are often not reported spontaneously. Thus, the parents must be directly asked about recurring symptoms in infancy and early childhood, since they might have been forgotten or interpreted differently.

### Motion sickness

Motion sickness was reported in 51% of our patients. The association of childhood migraine syndromes and motion sickness was reported previously, ranking between 43 and 50% (22, 46, 47) and in adults between 50 and 67% (48). The overlapping pathomechanisms of motion sickness and migraine remain controversial and are being addressed with an increasing number of studies (48–50).

### Family history of migraine

A positive family history of migraine (relatives of first or second grade) was found in 65.2%. A positive family history for periodic syndromes and/or migraine had been reported previously (42, 43, 51, 52). Parents of children with vertigo/dizziness should always be asked about this.

### Central ocular motor signs

Central ocular motor signs were found in 24% of our cohort, most frequently saccadic pursuit (18%). The only group without COMS was the group with BPV (as defined by the diagnostic criteria). Since vertical saccadic pursuit matures in late adolescence (19), it was not considered pathological, whereas horizontal smooth pursuit functions mature by the age of 7 years (20). Smooth pursuit eye movement abnormalities (tested by electronystagmography) in young patients presenting with vestibular symptoms were reported to be more frequent in children (45%) than in adolescents (26%) (2). The immaturity of the ocular motor system and/or central vestibular dysfunction are possible explanations (2). Marcelli et al. (53) found positional nystagmus in 44% of the children with migraine and vestibular symptoms (*n* = 22, aged 7–13 years), and post headshaking nystagmus in 31% (in one it had resolved). In the group of 18 migraineurs without vestibular symptoms (aged 8–13 years), he reported 33% with positional nystagmus and 33% with post headshaking nystagmus (53). Although we routinely perform the provoking maneuver with headshaking twice in our patients (by the doctor and by the orthoptic assistant), we found lower rates of abnormalities. One reason for this might be that we consider up to 4 nystagmus beats after headshaking as normal. Minor COMS were found to occur in the attack-free interval in more than 60% of adult patients with VM (9). Radtke et al. found ocular motor abnormalities in VM at the initial presentation in 15% of patients and in the follow-up examination in 41% (mean time of 9 years) (54). Our lower percentage might be in part explained by the fact that we did not consider isolated vertical saccadic pursuit to be a pathological finding in our patient cohort, although it is the most frequent central ocular motor sign described in adult patients (48%) (9). Another reason might be the increase of COMS with time as shown by Radtke et al. (54).

### Laboratory testing

The SVV deviated in 11% of our patients. There are no other studies of children with which our results could be compared. Adults have been reported to show a subclinical deviation of the SVV if they have a primary headache (tension-type and migraine) as opposed to patients without headache, but the values of deviation mentioned are still in a range we would consider normal (55).

We found 21% of pathologic results by caloric testing. Marcelli et al. reported a bilateral weakness in 25% and a unilateral weakness in 19% of childhood migraineurs with vestibular symptoms (*n* = 22), a bilateral weakness in 33% and a unilateral weakness in 17% of migraineurs without vestibular symptoms (*n* = 18) (53). Chang et al. examined 20 children with BPV (5–15 years, mean age 9 years) and compared them with 20 age- and sex-matched healthy children. They found abnormal responses in seven patients with BPV (35%) and normal responses in all healthy children (56). In contrast, O’Reilly et al. reported no objective peripheral vestibular abnormalities in 32 patients with migraine-related vertigo and BPV (*n* = 132) who presented to a tertiary care vestibular and balance laboratory (57). Boldingh et al. reported that 16% of adults with VM have pathologic caloric test values (*n* = 37) and 15.6% of patients with migraine but without vertigo (*n* = 32) (58). Shin et al. documented abnormal caloric test results in 25% of adult patients with dVM (*n* = 76) (59). Radtke et al. found 5% of patients had unilateral canal paresis (*n* = 38) at the initial testing, and 16% at follow-up (*n* = 45). Overall, the reports vary; methodological differences might play a role in these discrepancies, and the pathophysiology of these findings remains controversial.

Cervical vestibular evoked myogenic potentials were available for 47 patients; these were pathologic in 38%. Marcelli et al. reported bilaterally absent VEMP in 12% of children with migraine and vestibular symptoms (*n* = 22) and in 17% of children with migraine without vestibular symptoms (*n* = 18) (53). The data on children seem to be insufficient to allow us to make a statement about their usefulness for diagnosing VM. The use of VEMP in adult patients for differentiating VM from Menière’s disease is currently under discussion (60, 61).

Posturography was abnormal in nearly half of our patients. O’Reilly et al. reported minor abnormalities of static balance in 58% of 39 children with BPV, migraine, or another migraine equivalent (62). In our cohort, patients without clinical symptoms of a somatoform component also showed a pattern characteristic of phobic vertigo (increased postural sway at baseline with paradoxical improvement with eyes closed). This should alert us to the risk of these children developing SV in the future.

A limitation of our study was the selected cohort of patients. We are a tertiary center and receive patients who have been evaluated and often treated previously. On the other hand, this provides us with a longer case history to rely on. While the retrospective character of the study restricts the acquisition of complete data, it permits reevaluation of the patients on follow-up. These data need to be confirmed in prospective studies in order to evaluate the utility of the diagnostic criteria that have been mainly established for adults, along with the clinical outcome. Children might manifest the diagnostic migraine features later. A strength of our study is the large number of patients, all of whom were examined by the same team and received a standard work-up.

## Conclusion

The new diagnostic criteria for VM are well established and extremely important for clinical research. However, they are based mainly on adult data. Children and adolescents, in contrast, have a shorter medical history to establish a clinical diagnosis. Depending on their age, they might have difficulties to precisely describe their symptoms. Our data show that there is a group of patients who cannot be cataloged as having VM, although migraine might be the most reasonable explanation for their vertigo and/or dizziness. The current strict criteria are necessary to adequately compare study cohorts, but in the daily practice a therapeutic approach based on the most reasonable diagnosis is often used to minimize the fears of the parents and their children, to avoid interrupting leisure time and school activities, and – very importantly in our experience – to prevent the development of a secondary SV.

## Author Contributions

Thyra Langhagen designed the study, realized the data acquisition, analysis, and interpretation, drafted the initial manuscript, revised the manuscript, and approved the final manuscript as submitted. Nicole Lehrer contributed to the data acquisition and interpretation, revised the manuscript, and approved the final manuscript as submitted. Ingo Borggraefe and Florian Heinen contributed to the interpretation of the data, revised the manuscript, and approved the final manuscript as submitted. Klaus Jahn contributed to the data acquisition, analysis, and interpretation, critically reviewed the manuscript, and approved the final manuscript as submitted.

## Conflict of Interest Statement

The authors declare that the research was conducted in the absence of any commercial or financial relationships that could be construed as a potential conflict of interest.

## Abbreviations

BPTI, benign paroxysmal torticollis of infancy; BPV, benign paroxysmal vertigo; COMS, central ocular motor signs; cVEMP, cervical vestibular evoked myogenic potentials; dVM, definite vestibular migraine; ICHD-3 beta, International Classification of Headache Disorders, 3rd edition (beta version); oM, other migrain syndromes; pVM, probable vestibular migraine; sVM, suspected vestibular migraine; SVV, subjective visual vertical; vHIT, video head-impulse test; VOR, vestibular ocular reflex.
